# Effects of EOGO in Metakaolin-Based Geopolymer

**DOI:** 10.3390/ma18163864

**Published:** 2025-08-18

**Authors:** Chaewon Lee, Hoyoung Lee, Jinwoo An, Boo Hyun Nam

**Affiliations:** 1 Department of Civil Engineering, College of Engineering, Kyung Hee University, Yongin 17104, Republic of Korea; cindy12569@khu.ac.kr (C.L.); hoyounglee@khu.ac.kr (H.L.); 2 Department of Civil Engineering, College of Engineering and Computer Science, The University of Texas at Rio Grande Valley, Edinburg, TX 78539, USA; jinwoo.an@utrgv.edu

**Keywords:** geopolymer, metakaolin, graphene oxide, EOGO, mechanical properties, void content, setting time, rheology

## Abstract

Geopolymer concrete uses a geopolymer binder instead of traditional Portland cement; thus, it reduces carbon emissions by a significant amount. In this study, Edge-Oxidized Graphene Oxide (EOGO), a carbon-based nanomaterial, was added into a metakaolin-based geopolymer, and its effect on the mechanical and rheological properties of the mixture was investigated. EOGO was added into the mixture at 0% (control), 0.1%, 0.5%, and 1% of the metakaolin mass. Several experiments were conducted to characterize the properties of the metakaolin–EOGO (MKGO) geopolymer, including its compressive strength, free–free resonance column (FFRC), void content, water absorption, setting time, flow, and rheology. It was found that the compressive strength and stiffness showed their maximum values and the void content was minimized at 0.1% EOGO. In addition, as the EOGO addition rate increased, the setting time tended to shorten, and the fluidity tended to decrease. This suggests that 0.1% EOGO is the most optimal content in metakaolin paste. This study confirms that EOGO is an additive material that can improve the performance of metakaolin-based geopolymers and presents opportunities for the development of sustainable construction materials through optimization of EOGO addition.

## 1. Introduction

As climate change and environmental pollution issues have become increasingly serious worldwide, reducing carbon emissions in the construction industry is recognized as an important task. In particular, carbon dioxide (CO_2_) emissions generated during the production process of Portland cement are a significant burden on the environment, accounting for approximately 5–8% of the global human-made carbon emissions [1,2,3]. The limestone calcination process releases huge amounts of carbon dioxide, with about one ton of carbon dioxide being emitted per ton of cement produced from fossil fuel combustion [4]. The massive amount of CO_2_ produced during cement manufacturing is mainly due to the thermal decomposition of limestone and the combustion of fossil fuels, which are some of the main factors aggravating air pollution along with the consumption of energy resources. Geopolymers are attracting attention as an alternative to solve these carbon dioxide emission problems and develop carbon-neutral construction materials. Geopolymers are low-carbon, eco-friendly building materials produced through the alkali activation of industrial by-products or natural materials based on aluminosilicate. During the production process of 1 kg of geopolymer cement, only 0.18 kg of CO_2_ is emitted, which is only about 20% of that of ordinary Portland cement, and research results show that the CO_2_ emissions during the production process of geopolymer concrete are reduced by about 26% to 45% compared to those for ordinary concrete [5].

Therefore, geopolymers have potential as sustainable construction materials that can replace the existing Portland cement and are considered one of the key technologies for realizing a carbon-neutral society.

Geopolymerization is a process in which aluminosilicate raw materials react with an alkaline activator to form Al-Si hydrates, which condense and harden [6]. Any pozzolanic material containing silica and alumina and readily soluble in alkaline solutions can be used in the geopolymerization process [7]. In addition to geopolymerization, a pozzolanic reaction forming calcium alumina hydrate (CAH) and calcium silica hydrate (CSH) is also responsible for the long-term strength development of geopolymers [8]. The calcium silicate hydrate (CSH) compound is responsible for bonding between the grains and strength development [9]. Geopolymers are being studied as construction materials and grout materials because their production requires significantly less energy and emits significantly less CO_2_ than that of cement, and they exhibit excellent performances such as high strength and chemical resistance [10,11].

Metakaolin (MK), an aluminosilicate raw material, is produced by heating kaolin, one of the clay minerals naturally abundant in the Earth’s crust, at a temperature of 600 °C for 1 to 12 h [12]. Many studies have demonstrated that metakaolin-based geopolymers have excellent mechanical properties and long-term durability [13,14,15]. Various additives, such as nanomaterials and fibers, are used to improve the performance of geopolymers. Recent advances in nanotechnology have demonstrated that graphene oxide (GO) can improve the mechanical properties when used as an additive in geopolymers [16,17,18]. According to a study by Ho et al. (2021) [19], when pure GO was added to geopolymer mortar at 0.05% by weight (wt%) of the aluminosilicate raw materials, the compressive strength of the mixture was approximately 16%, 15%, and 14% higher than that of the mixture without GO at 7, 28, and 56 days, respectively. However, when producing GO using the existing Hummers’ method, the process involves multiple steps, including strong oxidation, extensive purification, and ultrasonication-assisted exfoliation. These steps require considerable chemical inputs, energy, and time, which contribute to high production costs [20]. This limits its practical use in large-scale applications such as construction.

As an alternative, Edge-Oxidized Graphene Oxide (EOGO) has been developed and is expected to be practical for mass use in the construction industry because it is produced using a simple and cost-effective ball milling process. Research on the use of EOGO as an additive to cement or concrete is also actively being conducted [21], and the microstructure and chemical composition of EOGO have been investigated to analyze the effects of EOGO on the mechanical properties of cement paste and mortar mixtures [22,23,24,25,26]. In a study by Cho et al. (2021) [27], the effect of EOGO on the flexural fatigue behavior of concrete composites was investigated. The results showed that EOGO reduced the bending deformation and increased the stiffness during their fatigue life. 

Although research on applying EOGO to cement and concrete is actively under way, there is no known prior research on applying it to geopolymers. Given the need for stable strength and durability in geopolymer materials for them to replace conventional cement, it is essential to explore additives like EOGO that can improve their performance. Recent studies [28,29] have also highlighted the role of nanomaterials in modifying the microstructure of clay-based geopolymers. However, most work remains qualitative or lacks macro-scale experimental validation. This study contributes to filling that gap.

In this study, pastes and specimens were prepared by adding EOGO to MK to analyze the effect of various contents of EOGO on a metakaolin-based geopolymer. EOGO was added at 0.1%, 0.5%, and 1% of the MK mass, and the resulting specimens were compared with the control paste or specimens without additions. To investigate the various properties of metakaolin–EOGO (MKGO), such as its mechanical properties and fluidity, a compressive strength test, free–free resonance column (FFRC) test, void content test, water absorption test, setting time test, flow test, and rheology test were performed. Through these evaluations, this study aimed to verify whether EOGO can serve as an effective additive for enhancing the mechanical, microstructural, and rheological properties of metakaolin-based geopolymers, thereby expanding the applicability of nanomaterials in producing sustainable construction materials.

## 2. Materials and Methods

### 2.1. Materials

#### 2.1.1. Metakaolin

Since metakaolin (Nycontech in Asan, South Korea) is obtained by calcining kaolinitic clay, a natural material, at a temperature of approximately 600 °C, it produces much less carbon dioxide than the cement production process, in which calcium carbonate is decomposed at 1450 °C. The chemical composition and physical properties of metakaolin are given in Table 1. Metakaolin (MK) is a pozzolanic material containing 49.1% silica (SiO_2_) and 43.2% alumina (Al_2_O_3_). The mean particle size of metakaolin is 11 μm. Compared to the particle sizes of cement and fly ash, which are 10–50 and 10–30 μm, respectively, it can be seen that it has very fine particles.

#### 2.1.2. Edge-Oxidized Graphene Oxide (EOGO)

**Figure 1:**
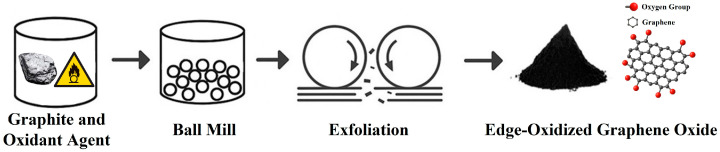
Process of EOGO production.

Edge-Oxidized Graphene Oxide (EOGO), used as an additive, is a type of reduced graphene oxide (GO) in which oxygen-containing functional groups are distributed mainly at the edges of the sheet. The existing method of producing GO involves a chemical oxidation technique such as Hummers’ method that uses strong oxidizers and concentrated acids [31]. In contrast, EOGO is synthesized via a mechanochemical ball milling process. If too weak a shear force is applied during the production of EOGO, the graphene layers will not be sufficiently separated, and the oxidation reaction will not occur properly. Conversely, if too strong a shear force is applied, the structure will be damaged and the particles will be crushed, resulting in the loss of the graphene’s properties. This method involves the ball milling of graphite powder with a mild oxidizer under precisely controlled conditions that optimize the shear force while minimizing the impact force [26]. Figure 1 describes the process of EOGO manufacturing.

**Figure 2:**
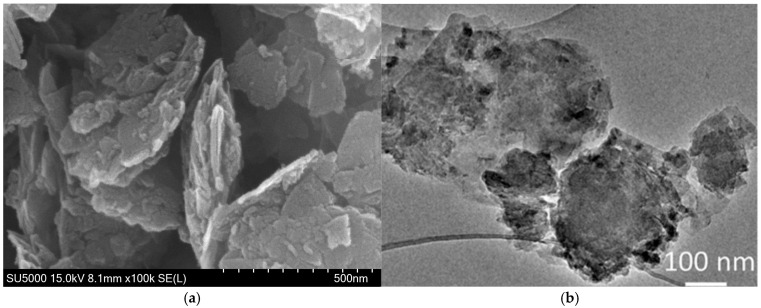
Material characterization of EOGO: (**a**) SEM; (**b**) TEM [26].

The characteristics of EOGO were investigated using Scanning Electron Microscopy (SEM), Transmission Electron Microscopy (TEM), and Attenuated Total Reflectance–Fourier Transform Infrared (ATR-FTIR) spectroscopy. SEM and TEM measurements conducted using JEOL instruments, and ATR-FTIR spectroscopy performed using a Bruker Corporation instrument. The results of SEM and TEM for EOGO are illustrated in Figure 2a and Figure 2b, respectively. The SEM image of EOGO (Figure 2a) shows a wrinkled sheet morphology and a noticeably rough surface, which is due to mechanical deformation induced by the ball milling process. The TEM image (Figure 2b) clearly shows the multilayer structure of EOGO with overlapping graphene.

**Figure 3:**
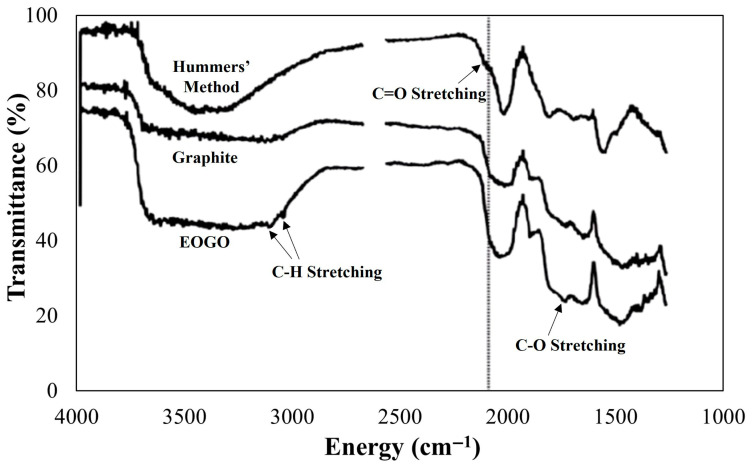
ATR-FTIR spectroscopy (Hummers’ method-produced GO, graphite, and EOGO) [26].

Figure 3 compares the results of ATR-FTIR spectroscopy of graphite, GO (Hummers’ method), and EOGO. In the graph, the *x*-axis represents the energy of the light, and the *y*-axis represents the degree of light penetration. A lower y value means that oxygen radicals of that wavelength are present and light is absorbed. Due to the strong oxidation process, GO has various oxygen groups such as C-H, COOH, and C-O evenly distributed across its entire surface. In comparison, EOGO does not have as strong a C-O or COOH peak as GO, with its main peaks related to C-H and C-O. When oxidation occurs at the terminal functional groups present at the edges of graphene, C-H, C-O, and C=O functional groups are mainly generated. Therefore, our analysis of EOGO can confirm that oxidation occurs mainly at the edges of the GO.

EOGO was procured from Garmor, Inc. (Orlando, FL, USA), and the chemical composition of EOGO is given in Table 2.

### 2.2. Preparation of MKGO Paste and Specimens

**Figure 4:**
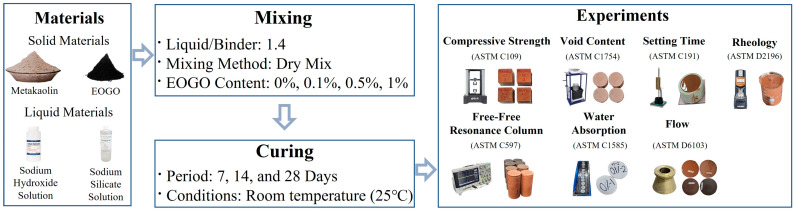
Experimental design.

The materials used in this experiment were divided into solid materials and liquid materials. The solid materials included metakaolin and EOGO, while the liquid materials included a sodium hydroxide (NaOH) solution called SHS (Daemyeong in Osan, South Korea), and sodium silicate (Na_2_SiO_3_) solution called SSS (Samchun in Seoul, South Korea). NaOH acted as an alkaline activator to dissolve metakaolin, an aluminosilicate raw material, and promote the reaction. Na_2_SiO_3_ played a role in supplying additional silica required in the geopolymer reaction. There were a total of four mixtures with different EOGO contents. The mixture design is summarized in Table 3. The liquid-to-binder ratio (L/B) was 1.4 for all the mixtures. The liquid consisted of SSS and SHS, and the binder consisted of metakaolin. The specimen was made using the Dry Mix method. The Dry Mix method first involves the mixing of the solid materials (MK and EOGO), and then mixing with the liquid solution. For experiments using cured specimens, the specimens were prepared by curing them at a room temperature of 25 °C in accordance with the ASTM standards for each experiment. The experimental design is given in Figure 4. For reference, a paste ID was assigned to each MKGO paste, ‘‘MKGO-X”, where X indicates the content of EOGO. The paste containing 0% EOGO was set as the control paste (CP), and the specimen manufactured with the CP was set as the control specimen (CS). The EOGO contents were selected based on previous studies involving the incorporation of EOGO in cementitious materials. A dosage range of 0.01–1.0 wt% is commonly regarded as the typical range for using GO-based materials as additives when aiming to improve the mechanical or microstructural performance of cementitious or geopolymer matrices [21,26]. The testing methods employed in this study were compressive strength, free–free resonance column (FFRC), void content, water absorption, setting time, flow, and rheolometer tests.

### 2.3. Test Methods

#### 2.3.1. Compressive Strength

Compressive strength tests were performed to evaluate the effect of EOGO on the compressive strength of metakaolin-based geopolymers. The compressive strength tests were performed according to ASTM C109 [32], and the specimen size was 50 × 50 × 50 cubic mm. All specimens were cured at a room temperature of 25 °C for 7, 14, and 28 days. Three specimens made from each mixture were tested for their compressive strength, and the results were averaged. The compressive strength test was performed using a strain control Universal Testing Machine (JH Tech in Yongin, South Korea)), and stress–strain curves were obtained.

#### 2.3.2. Free–Free Resonant Column (FFRC)

**Figure 5:**
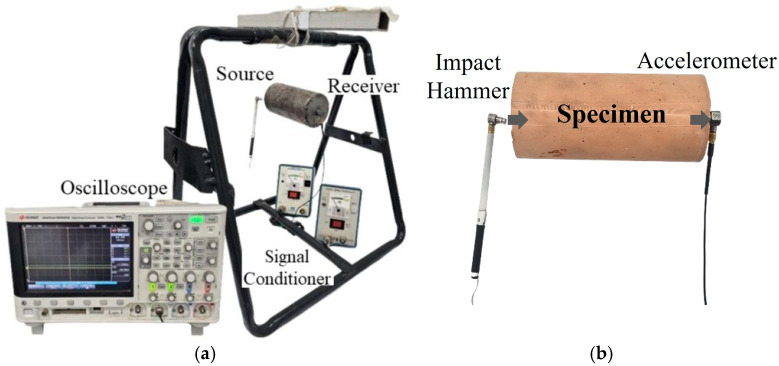
Free–free resonance column test (FFRC). (**a**) Experimental setup; (**b**) specimen setup.

FFRC tests were performed to evaluate the effect of EOGO on the stiffness of metakaolin-based geopolymers. The FFRC test was carried out according to ASTM C597 [33], and the test was performed on cylindrical specimens with a diameter of 50 mm and a height of 100 mm. The experimental and specimen setups are depicted in Figure 5. To evaluate the effect of the curing time, the test was performed on specimens cured for 7, 14, and 28 days. When one side of the specimen was hit by an impact hammer, resonance occurred in the specimen. The accelerometer on the other side measured the response of the specimen and determined the resonance frequency. The time-domain signal obtained from the receiver was processed using the FFT (Fast Fourier Transform) using MATLAB (R2024a), and the peak frequency was determined, which was the resonance frequency. This frequency reflected the material’s stiffness based on wave propagation theory, as discussed in related studies [34]. The stiffness, Young’s Modulus (E), was determined using Equations (1)–(3).

(1) λ=2L

(2) $$V_{c}=f_{r}\times \lambda$$

(3) $$E=\rho \times {V_{c}}^{2}$$

where λ = the wavelength, L = the length of the specimen, $f_{r}$ = the resonance frequency, and ρ = the density of the specimen (g/mm^3^).

#### 2.3.3. Void Content

A void content test was performed to evaluate the effect of EOGO on pore formation. In accordance with ASTM C1754 [35], the experiments were conducted on cylinders manufactured with a shape 100 mm in diameter and 50 mm in height and cured for 28 days. After measuring the oven-dried weight and the weight when immersed in water, the void content was calculated according to Equation (4).

(4) $$Void\;Content=[1-(\frac{k\times \left(A-B\right)}{P_{w}\times T\times W\times L})\times 100]$$

where *A* = the dry mass of the specimen (g), *B* = the submerged mass of the specimen (g), *T* = the thickness (mm), *L* = the average length of the specimen (mm), *W* = the width (mm), and k = the correction factor.

#### 2.3.4. Water Absorption

A water absorption test was performed to evaluate the effect of EOGO on the water absorption rate of the specimens. The experiments were conducted on cylinders with a diameter of 100 mm and a height of 50 mm, in accordance with ASTM C 1585 [36]. After curing the specimens for 28 days, we applied waterproof paint to the sides and top of the specimens so that only the bottom side absorbed water. The weight of the specimens was measured at set intervals, and the absorption rate *I* was calculated according to Equation (5).

(5) $$I=\frac{m_{t}}{a\times d}$$

where I  = the absorption (mm), $m_{t}$ = the change in the specimen mass in grams at time *t* (g), a  = the exposed area of the specimen (mm^2^), and d  = the density of the water (g/mm^3^).

#### 2.3.5. Setting Time

A setting time test was performed to evaluate the effect of EOGO on the initial and final setting times. The paste was put in a mold and a Vicat needle test was performed in accordance with ASTM C191 [37]. When the needle was dropped, the point when the penetration was 25 mm was defined as the initial setting time, and the point when the penetration was 0 mm was defined as the final setting time.

#### 2.3.6. Flow

Flow tests were performed to evaluate the effect of EOGO on the fluidity of the paste. The flow tests were conducted by filling a cylinder frame with a diameter of 760 mm and a height of 150 mm with paste and lifting it, in accordance with ASTM D6103 [38]. We measured the diameter of the spread paste eight times and calculated the average.

#### 2.3.7. Rheology

A rotary viscometer test (Brookfield Engineering in Middleboro, MA, USA) was performed to evaluate the effect of EOGO on the rheological properties of the paste. This rheology test obtained the apparent viscosity by rotating a vane spindle. The test procedure followed ASTM D2196 [39]. The viscosity as a function of the shear rate was calculated, and the viscosity of the pastes based on the EOGO addition rate was compared.

## 3. Results

### 3.1. Effects of EOGO on Mechanical Properties of Metakaolin-Based Geopolymer

**Figure 6:**
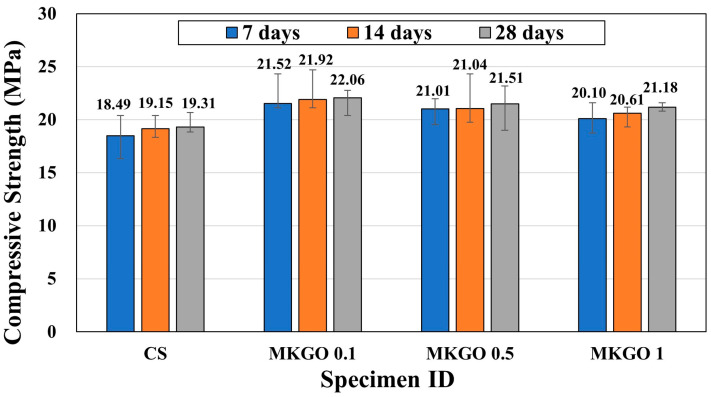
Compressive strength of specimens cured for 7, 14, and 28 days.

Figure 6 shows the results of the compressive strength test for specimens cured for 7, 14, and 28 days. Overall, the compressive strength increased as the curing period increased, but with a slight increase. Previous studies [40,41] reported that the compressive strength of metakaolin geopolymers tended to increase as the curing period increased. This trend occurs because as the curing period increases, more cross-linking of the Si-O-Al bonds occurs within the geopolymer, which makes the structure denser, reduces its porosity, and increases its strength. Compared to the 7-day curing period, at 1% EOGO (MKGO 1), the specimen of MKGO 1 cured for 28 days showed the maximum increase, with a 5.37% increase in the compressive strength compared to that of the specimen cured for 7 days.

The specimens with added EOGO exhibited higher compressive strength than the control under all curing conditions. The specimen with 0.1% EOGO (MKGO 0.1) showed the highest compressive strength. When compared with the control (CS), at 28 days of curing, the strength of MKGO 0.1 was increased by 14.2%. However, interestingly, when the EOGO content was 0.5% or 1%, the compressive strength decreased compared to that with 0.1% EOGO. It is known that uniformly dispersed EOGO has the effect of increasing the internal microstructure of cement [24]; however, nanoparticles have a tendency to aggregate, and this aggregation can hinder the nanoparticles from performing well in mixtures [42]. Excessive EOGO addition may reduce the compressive strength due to an agglomeration effect and increase in micro voids [43]. When EOGO is added in a small amount of 0.1%, the EOGO is uniformly dispersed, but when the EOGO content is 0.5% or 1%, the dispersibility decreases due to agglomeration compared to when EOGO is added in a small amount of 0.1%, forming an uneven microstructure. Agglomerated regions interrupt the continuity of the sodium aluminosilicate hydrate (N-A-S-H) gel network and reduce the matrix cohesion. Similar mechanisms have been revealed in prior studies [44] that systematically compared uniformly dispersed and re-agglomerated GO in cement pastes using SEM, MIP, and BET, showing that uniform dispersion effectively reduces the presence of macropores, refines the pore structure, and increases the hydration products, whereas re-agglomeration hinders the performance. A non-uniform microstructure may cause voids or defects within the specimen, which may reduce the strength-enhancing effect.

**Figure 7:**
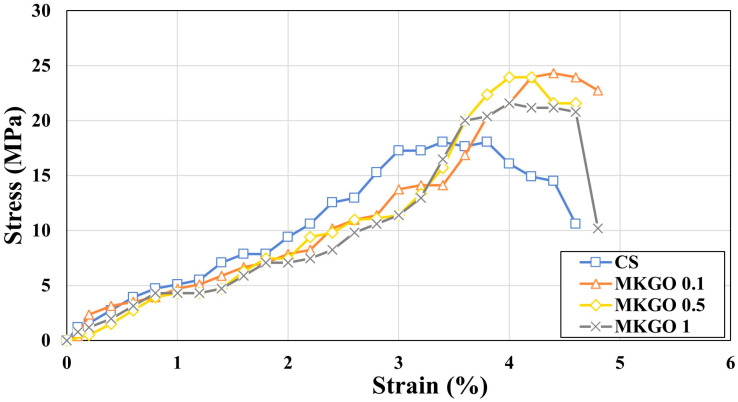
Stress–strain curve of specimens cured for 14 days.

Figure 7 shows the stress–strain graphs of the specimens. In a stress–strain graph, the slope of the curve represents the stiffness of the specimen. Compared to the control (no EOGO), the slope is shallower for the specimens with EOGO. It can be seen that the stiffness of the specimen decreased with increasing EOGO. The peak point of the curve represents the compressive strength, while the area under the failure curve represents the toughness of the specimen. The toughness values of CS, MKGO 0.1, MKGO 0.5, and MKGO 1 were 43.82, 50.32, 46.33, and 50.23, respectively, and when compared with those of the CS, those of MKGO 0.1, MKGO 0.5, and MKGO 1 increased by percentages of 14.82%, 5.73%, and 14.64%, respectively. It is believed that the reason for increased toughness with a higher content of EOGO is that despite higher deformation, EOGO may act as a “bridging” fiber that holds together micro-cracks within the skeleton. According to previous studies [45,46,47], cross-linked GO sheets can be generated even at very low dosages of carbon nanomaterials. When EOGO is well dispersed within the geopolymer matrix, these nanosheets may form a connected network structure that holds the particles and micro-cracks together under dynamic loading, which can contribute to improved stiffness, as observed in the test results. However, at higher EOGO contents, particle agglomeration or uneven dispersion may occur, which can hinder the formation of a uniform load-bearing structure and partially offset the stiffness gains. This may explain the slight decrease in stiffness observed in the 1% EOGO sample. This mechanism is referred to as the bridging or crack bridging effect in some studies [45,48], contributing to the energy absorption and toughness, particularly under dynamic loading conditions.

**Figure 8:**
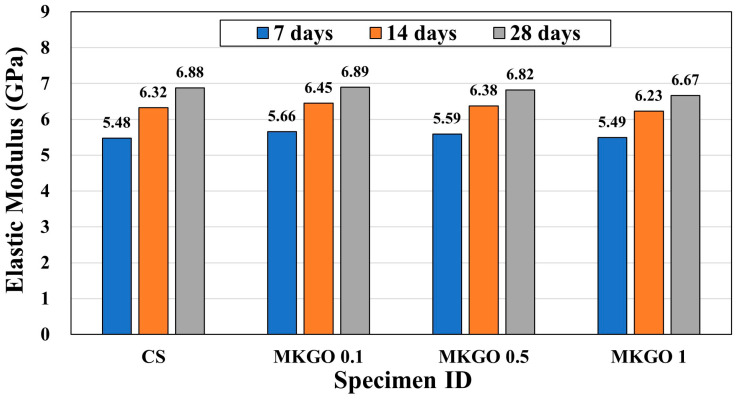
Elastic modulus of specimens obtained using FFRC test.

The results of the free–free resonance test are shown in Figure 8. FFRC testing was performed on specimens (CS, MKGO 0.1, MKGO 0.5, and MKGO 1) cured for 7, 14, and 28 days. All specimens showed a tendency for their elastic modulus to increase as the curing period increased. Compared to the stiffness of the control specimen after 7 days, the stiffness of MKGO 0.1 and MKGO 0.5 increased by 3.3% and 4.5%, respectively. This could be because EOGO inhibits crack propagation and reinforces the microstructure within the geopolymer framework. At the nanoscale, EOGO acts to strengthen the bonds between particles within the structure, which increases the elastic modulus by strongly resisting deformation. However, excessive addition of EOGO may lead to decreased dispersibility and coagulation. In MKGO 1, the stiffness was reduced by 3.1% compared to that of the CS in the 28-day specimens.

### 3.2. Effects of EOGO on Void Content of Metakaolin-Based Geopolymer

**Figure 9:**
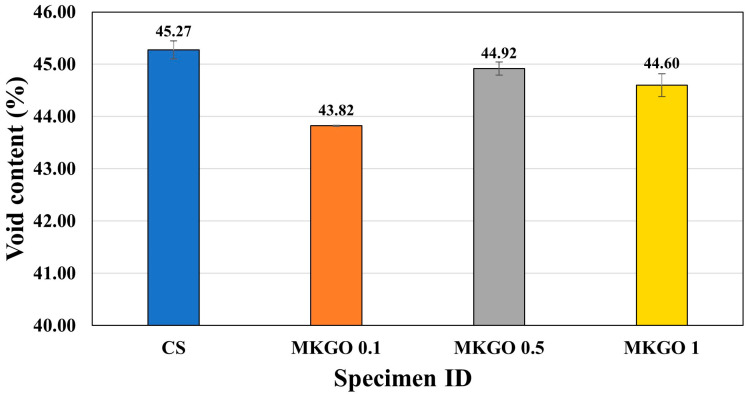
Void content of specimens cured for 28 days.

Figure 9 shows the results of the porosity test of specimens cured for 28 days. In all specimens with added EOGO, the void content tended to decrease compared to that of the CS. For cement hydration, EOGO has the function of reducing the number of large capillary pores and refining the pores [43]. Similar improvements in the mechanical properties have also been reported in GO and rGO composites. In particular, GO’s high dispersibility and abundant functional groups contribute to chemical bonding with cement hydration products, resulting in matrix densification and enabling bonding interactions at the nano–microscale. In the case of rGO, the enhancements are primarily attributed to its high physical strength and residual functional groups that participate in bonding. These mechanisms were confirmed through SEM-EDX analysis in previous studies [47]. Furthermore, Liu et al. [16] demonstrated via FTIR, XRD, and SEM analyses that even low-dosage additions of GO or rGO can reduce the pore volume and accelerate geopolymerization in fly ash-based geopolymers, thereby enhancing structural densification and refining the microstructure. Consequently, it seems that nano-sized EOGO fills nano-pores and reduces the porosity and densifies the paste. MKGO 0.1 had the largest reduction in its void content, with a 3.2% reduction compared to the CS. As discussed earlier, at 0.1% of EOGO, the dispersion of EOGO within the specimen is high (probably no agglomeration), so the pores are filled uniformly. In addition, evenly dispersed EOGO with oxygen-containing functional groups can promote the formation of nucleation sites and the formation of a more cross-linked N-A-S-H gel, leading to a reduction in the void content. In contrast, when EOGO is added at 0.5% or more, the pore filling effect becomes minimal. EOGO has a large surface area and a plate-like structure, so it tends to aggregate easily. If excessive amounts, such as 0.5% and 1% EOGO, are added, they will probably clump together and disperse unevenly.

### 3.3. Effects of EOGO on Water Absoption of Metakaolin-Based Geopolymer

**Figure 10:**
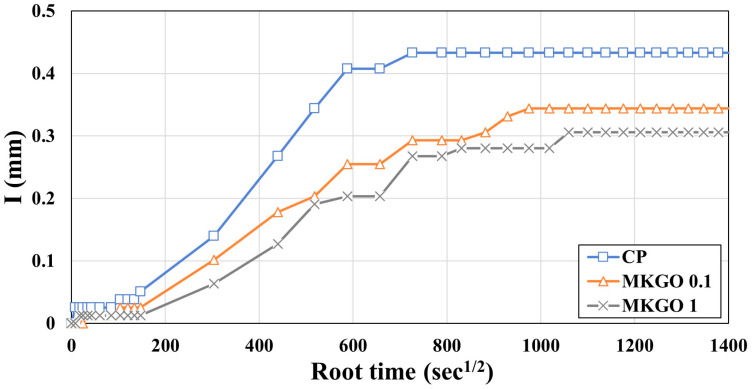
Absorption test results for specimens after 28 days of curing.

The results of the water absorption test of specimens cured for 28 days are shown in Figure 10. In Figure 10, I (cumulative water absorption) represents the depth of the water penetration. The root time (√t) was used because capillary absorption follows a diffusion-based mechanism, where the absorption is proportional to the square root of time in the early stages. The results for the MKGO 0.5 specimen were not plotted due to problems with cracks occurring during the experiment. In the specimens with added EOGO, both the absorption rate and absorption amount were reduced compared to those of the CS. While the water absorption rate of the CS was 0.43 mm, the water absorption rate of MKGO 0.1 and MKGO 1 decreased to 0.34 mm and 0.28 mm, respectively. The reason for the decrease in the water absorption is that EOGO refines the pores, thereby improving the resistance of specimens to water absorption. Also, as can be seen from the graph, it took the CS about 62 h to absorb 0.3 mm of water, while MKGO 0.1 and MKGO 1 took about 216 h and 156 h, respectively. Oxygen groups such as the carboxyl group (-COOH) and hydroxyl group (-OH) attached to EOGO have hydrophilic properties and instantly adsorb water molecules, reducing the rate of moisture diffusion. Previous studies further confirmed the hydrophilicity of EOGO, reporting a contact angle of approximately 88.3°, which is lower than the 90° threshold for hydrophilicity [49]. Additionally, when EOGO is dispersed and present within the structure, each particle is surrounded by oxygen groups, which act as a physical barrier for water particles to pass through. In particular, when the plate-like structure of EOGO is arranged horizontally, the capillary velocity is further reduced, which further reduces the moisture diffusion rate. This capillary absorption behavior can also be explained by diffusion-based mechanisms, particularly Fick’s law, in the early stages. While detailed modeling is beyond the scope of this experimental study, a more mechanistic approach to moisture transport can be found in a previous study as proposed by Cortis and Berti [50].

### 3.4. Effects of EOGO on Setting Time of Metakaolin-Based Geopolymer

**Figure 11:**
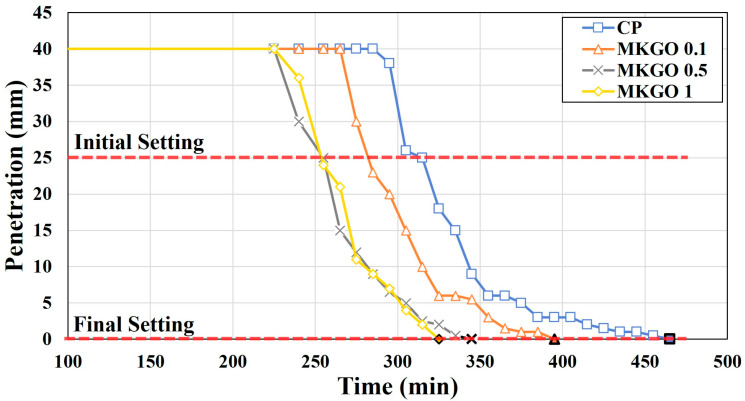
Setting times of geopolymer paste obtained using Vicat needle test.

The results of the test of the pastes’ setting time are shown in Figure 11. The initial and final setting times of a geopolymer represent critical stages in the geopolymerization process. The initial setting time is the time from mixing until the paste begins to lose its workability and starts to harden. The final setting time is the point at which the paste has completely lost its plasticity and has gained sufficient rigidity to resist minor pressure. The initial setting times of the CP, MKGO 0.1, MKGO 0.5, and MKGO 1 were 315 min, 280 min, 255 min, and 255 min, respectively. These results are shown in Table 4. The final setting times were 465 min, 395 min, 345 min, and 325 min, respectively. As the EOGO content increased, the initial and final setting times tended to shorten. When EOGO was added, the initial setting time was reduced from 11% to 19%, and the final setting time was reduced from 15% to 30%. On the other hand, when the content of EOGO increased from 0.5% to 1%, the initial setting time remained the same and the final setting time decreased by only 20 min. From the results of this experiment, it can be confirmed that the effect of shortening the curing time decreases when EOGO is added at 0.1% or more. This is because when EOGO is added at more than the appropriate amount, sufficient gel has already formed and this does not contribute to additional acceleration of the reaction rate.

### 3.5. Effects of EOGO on Rheological Properties of Metakaolin-Based Geopolymer

**Figure 12:**
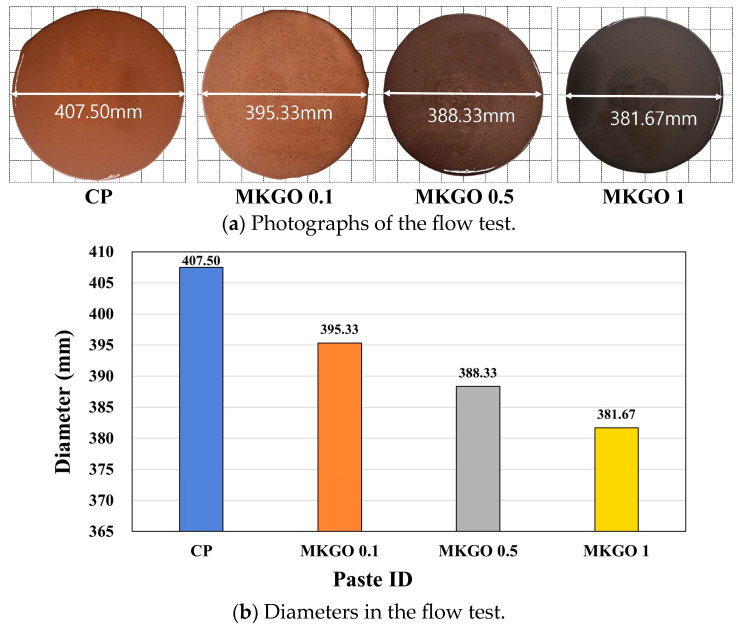
Flow test results for geopolymer paste.

Figure 12 shows the results of the flow test of the pastes. As seen in Figure 12a, as the EOGO content increased, the diameter of the spread paste gradually decreased. As shown in Figure 12b, the diameters of the pastes were measured as 407.50, 395.33, 388.33, and 381.67 mm, respectively. In the MKGO 0.1, MKGO 0.5, and MK 1 pastes with added EOGO, the diameters were reduced by 2.99%, 4.71%, and 6.33%, respectively, compared to that of the CP. EOGO has oxygen groups such as a carboxyl group (-COOH) and hydroxyl group (-OH), so it has a strong tendency to bind to water. Therefore, the viscosity increases and the diameter to which the paste spreads decreases because the moisture in the paste is adsorbed to the EOGO and the effective moisture content decreases. In addition, EOGO has a thin, plate-like structure, which increases the interparticle friction and internal resistance within the paste, causing an increase in the shear resistance and a decrease in fluidity.

**Figure 13:**
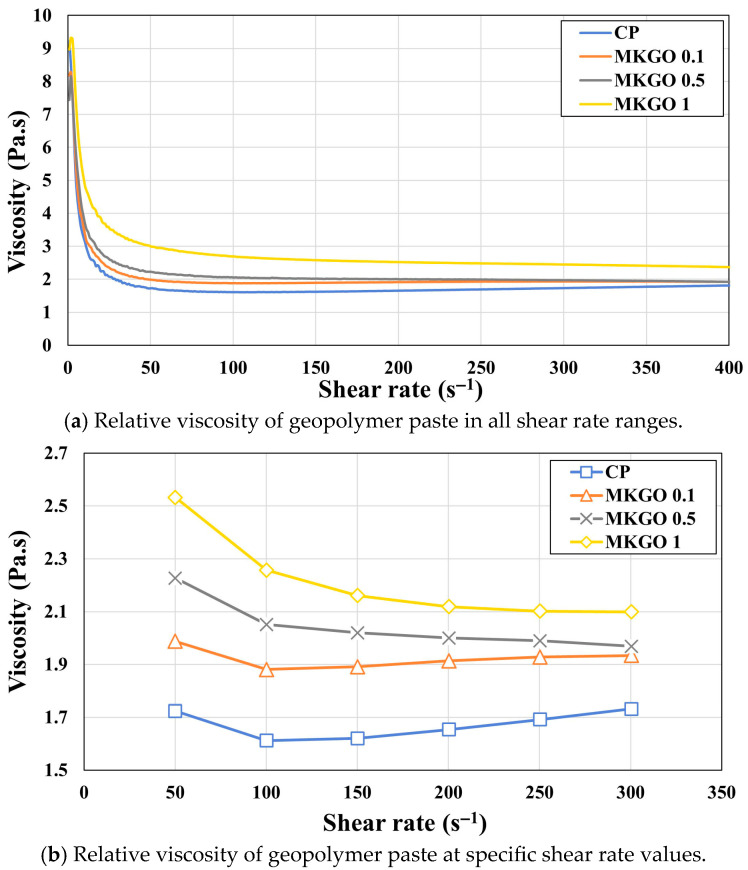
Relative viscosity of geopolymer paste obtained using rheology test.

The results of the rheology test of the pastes are shown in Figure 13. Figure 13a shows the viscosity of the pastes with different EOGO contents according to the shear rate, from a shear rate of 0 s^−1^ to a shear rate of 400 s^−1^, at which the viscosity became stable. A clear difference was seen in the viscosity when the shear rate increased in 50 s^−1^ increments from 50 s^−1^ to 300 s^−1^, as shown in Figure 13b. When the shear rate was 50 s^−1^, the viscosities were 1.724, 1.988, 2.227, and 2.532 Pa.s, respectively. It can be confirmed that the viscosity tended to increase as the EOGO content increased. At this time, the viscosity of MKGO 1 showed a result of a 45% increase compared to that of the CP. It is known that hydrophilic EOGO has a large surface area and can absorb water molecules on its surface, thereby reducing the free water content required for lubrication, which leads to a decrease in the fluidity of cement paste and an increase in its viscosity [51]. As the EOGO content increases, the free water in the paste decreases, which reduces the viscosity at the same shear rate. As can be seen in Figure 13b, in the CP without EOGO added, the viscosity decreased as the shear rate increased and then increased again above 100 s^−1^, but in all the pastes with EOGO added, the viscosity decreased or remained constant as the shear rate increased. Since EOGO has a negative charge in an aqueous solution, it reduces the collisions between particles when the shear rate increases, causing the viscosity to decrease or remain constant.

## 4. Discussions

In this study, the effect of EOGO on a metakaolin geopolymer was evaluated. Similarly to in previous studies [26,44] where the incorporation of EOGO into cement-based materials led to improved mechanical properties and pore refinement, the addition of EOGO to geopolymer composites in this study also resulted in enhanced strength and a densified microstructure. This suggests that EOGO provides comparable reinforcement effects in both cementitious and geopolymer matrices. The mixture with the best mechanical performance was MKGO 0.1. A 0.1% EOGO content was determined to be the optimal content as an additive because MKGO 0.1 exhibited the highest strength, stiffness, and toughness. Micro-pores, which have the greatest influence on the performance of geopolymers, was improved by EOGO holding micro-cracks together and filling micro-pores through two functions: the “bridging effect” and “pore filling effect.” These effects were minimal at EOGO levels above 0.5% due to coagulation through electrical interaction. In addition, evenly dispersed EOGO with oxygen-containing functional groups (MKGO 0.1) could promote the formation of nucleation sites and the formation of a more cross-linked N-A-S-H gel, leading a reduction in the void content and an increase in the mechanical properties. However, at high concentrations of EOGO (0.5% or more), agglomerated regions interrupt the continuity of the N-A-S-H gel network and reduce the matrix cohesion. Addressing this challenge through improved dispersion techniques or surface modification strategies is a promising direction for future research. Another effect of EOGO was that it decreased the water absorption of geopolymer specimens because EOGO refines the pores through its pore filling effect and the formation of a more cross-linked N-A-S-H gel, thereby improving the resistance of specimens to water absorption. And EOGO also shortened the setting time and reduced the fluidity of the paste because it absorbed more water.

## 5. Conclusions

In this study, EOGO was used as an additive in a metakaolin-based geopolymer. EOGO was added to the geopolymer mixture at four different percentages: 0%, 0.1%, 0.5%, and 1%. Room-temperature curing conditions were applied. Compressive strength and FFRC tests were conducted to evaluate the strength and stiffness. In addition, void content, water absorption, setting, flow, and rheology tests were performed to measure the durability of the geopolymer. The conclusions are as follows:

- Through compressive strength and FFRC tests, we found that the metakaolin-based geopolymer had higher strength and stiffness with an increased curing period. The strength and stiffness increased with the addition of EOGO, and the highest strength and stiffness were achieved at 0.1% EOGO due to the bridging effect, pore filling effect, and the formation of a more cross-linked N-A-S-H gel. Compared to the control specimen, 0.1% EOGO increased the strength by an average of 15% and the stiffness by 1.78%. However, at high concentrations of EOGO (0.5% or more), agglomerated regions interrupted the continuity of the N-A-S-H gel network and reduced the matrix cohesion.
- Void content measurement showed that nano-sized EOGO had the effect of filling the pores of geopolymers and reducing the void content. In particular, the greatest decrease was observed at 0.1% EOGO, at which the void content was 3.2% lower than that of the control specimen. EOGO levels of 0.5% and 1% had a higher void content than 0.1% EOGO, which was due to the creation of larger pores caused by the agglomeration and non-uniform dispersion of EOGO.
- Due to the high surface area and hydrophilic properties of EOGO due to the oxygen-containing functional groups on its surface, the free moisture in the paste was adsorbed by EOGO. This phenomenon shortened the setting time of the geopolymer paste and reduced its fluidity. In particular, the reduction effect was the greatest at 0.1% EOGO. These results suggest that a metakaolin geopolymer with EOGO is a good cement substitute that can reduce carbon dioxide emissions and that it has the potential to become a construction material with excellent performance through the optimization of the properties of the geopolymer through further research.
- Although this study focused on microstructural and short-term durability indicators such as the void content and water absorption, long-term durability aspects such as the drying shrinkage, sulfate resistance, and environmental degradation were not directly assessed. Future research should include these parameters to fully understand the aging behavior and service life performance of EOGO-enhanced geopolymer composites.

## Figures and Tables

**Table 1:** The chemical composition and physical properties of metakaolin [30].

Chemical Composition (%)				Physical Properties	
SiO_2_	Al_2_O_3_	Fe_2_O_3_	K_2_O + Na_2_O	Mean Particle Size (μm)	Specific Gravity
49.1	43.2	1.32	0.57	11	2.6

**Table 2:** Chemical composition of EOGO.

Oxygen	Non-Oxygen Composition						
	Carbon	Silicon	Sulfur	Potassium	Calcium	Chromium	Copper
5~10%	>99.8%	<40 ppm	<60 ppm	<5 ppm	<30 ppm	<125 ppm	<5 ppm

**Table 3:** Composition of metakaolin–EOGO (MKGO) composite mixture.

Paste ID	L/B Ratio	Metakaolin (g)	SSS (g)	SHS (g)	EOGO (%)	EOGO (g)
CP	1.4	838	838	335	0	0
MKGO 0.1	1.4	838	838	335	0.1	0.84
MKGO 0.5	1.4	838	838	335	0.5	4.19
MKGO 1	1.4	838	838	335	1	8.38

**Table 4:** Initial and final setting times of geopolymer paste.

Paste ID	Initial Setting Time (min)	Final Setting Time (min)
CP	315	465
MKGO 0.1	280	395
MKGO 0.5	255	345
MKGO 1	255	325

## Data Availability

The original contributions presented in this study are included in the article. Further inquiries can be directed to the corresponding author.
